# P2 receptor mRNA expression profiles in human lymphocytes, monocytes and CD34+ stem and progenitor cells

**DOI:** 10.1186/1471-2172-5-16

**Published:** 2004-08-03

**Authors:** Lingwei Wang, Sten Eirik W Jacobsen, Anders Bengtsson, David Erlinge

**Affiliations:** 1 Department of Cardiology, Lund University Hospital, S-221 85 Lund, Sweden; 2 Department of Hematopoietic Stem Cell Laboratory, Lund Center for Stem Cell Biology and Cell Therapy, Lund University Hospital, S-221 85 Lund, Sweden; 3 Department of Rheumatology, Lund University Hospital, S-221 85 Lund, Sweden

**Keywords:** P2 receptor, real-time PCR, lymphocytes, monocytes, CD34^+ ^cells, hematopoietic stem cells

## Abstract

**Background:**

Extracellular nucleotides (ATP, ADP, UTP and UDP) exert a wide range of biological effects in blood cells mediated by multiple ionotropic P2X receptors and G protein-coupled P2Y receptors. Although pharmacological experiments have suggested the presence of several P2 receptor subtypes on monocytes and lymphocytes, some results are contradictory. Few physiological functions have been firmly established to a specific receptor subtype, partly because of a lack of truly selective agonists and antagonists. This stimulated us to investigate the expression of P2X and P2Y receptors in human lymphocytes and monocytes with a newly established quantitative mRNA assay for P2 receptors. In addition, we describe for the first time the expression of P2 receptors in CD34^+ ^stem and progenitor cells implicating a potential role of P2 receptors in hematopoietic lineage and progenitor/stem cell function.

**Results:**

Using a quantitative mRNA assay, we assessed the hypothesis that there are specific P2 receptor profiles in inflammatory cells. The P2X_4 _receptor had the highest expression in lymphocytes and monocytes. Among the P2Y receptors, P2Y_12 _and P2Y_2 _had highest expression in lymphocytes, while the P2Y_2 _and P2Y_13 _had highest expression in monocytes. Several P2 receptors were expressed (P2Y_2_, P2Y_1_, P2Y_12_, P2Y_13_, P2Y_11_, P2X_1_, P2X_4_) in CD34+ stem and progenitor cells.

**Conclusions:**

The most interesting findings were the high mRNA expression of P2Y_12 _receptors in lymphocytes potentially explaining the anti-inflammatory effects of clopidogrel, P2Y_13 _receptors in monocytes and a previously unrecognised expression of P2X_4 _in lymphocytes and monocytes. In addition, for the first time P2 receptor mRNA expression patterns was studied in CD34^+ ^stem and progenitor cells. Several P2 receptors were expressed (P2Y_2_, P2Y_1_, P2Y_12_, P2Y_13_, P2Y_11_, P2X_1_, P2X_4_), indicating a role in differentiation and proliferation. Thus, it is possible that specific antibodies to P2 receptors could be used to identify progenitors for monocytes, lymphocytes and megakaryocytes.

## Background

Extracellular nucleotides (ATP, ADP, UTP and UDP) exert a wide range of biological effects in blood cells mediated by multiple ionotropic P2X receptors and G protein-coupled P2Y receptors [1-3]. So far, the P2Y family is composed of eight cloned and functionally distinct subtypes (P2Y_1_, P2Y_2_, P2Y_4_, P2Y_6_, P2Y_11_, P2Y_12_, P2Y_13_, P2Y_14_) [4,5]; the P2X family is composed of seven cloned subtypes (P2X_1_-P2X_7_) [6,7].

We have previously quantified P2 receptor mRNA expression in platelets (representing megakaryocyte expression), and demonstrated a selective expression of the ADP receptors P2Y_12 _and P2Y_1_, together with the ATP receptor P2X_1 _[8]. This is consistent with the clinical effect of the P2Y_12 _antagonist clopidogrel for the prevention of myocardial infarctions in patients with acute coronary syndromes [9,10]. However, virtually every hematopoietic cell is responsive to nucleotides [2]. Because effects as different as proliferation, differentiation, chemotaxis and release of cytokines are regulated by nucleotides, they could play a role in the atherosclerotic inflammatory process. Human lymphocytes, monocytes and macrophages constitute an important line of defence upon infection and exposure to inflammatory stimuli [11]. Circulating blood monocytes become activated, migrate to tissues, and undergo differentiation into macrophages during inflammation [12]. Monocytes have been shown to express several P2Y receptors and up-regulation of P2X_7 _receptor mRNA in monocytes has been observed upon cell differentiation to macrophages [13,14].

Although pharmacological experiments have suggested the presence of several P2 receptor subtypes on monocytes and lymphocytes, some results are contradictory [1,2]. Few physiological functions have been firmly established to a specific receptor subtype, partly because of a lack truly selective agonists and antagonists. This stimulated us to investigate the expression of P2X and P2Y receptors in human lymphocytes and monocytes with a newly established quantitative mRNA assay for P2 receptors [8,15]. In addition, we describe for the first time the mRNA expression of P2 receptors in CD34^+ ^stem and progenitor cells implicating a potential role of P2 receptors in hematopoietic lineage and progenitor/stem cell function.

## Results and Discussion

Our previous studies of P2 receptor mRNA expression in man with real-time PCR has shown a good resemblance with pharmacological and physiological experiments in vascular smooth muscle cells, endothelial cells and platelets [8,15]. It is therefore likely that our present mRNA findings in inflammatory, progenitor and stem cells are physiologically relevant. The lack of selective agonists and antagonists for most of the receptor subtypes combined with the absence of studies focused on several of the more recently cloned receptors makes the findings important. Furthermore, no pharmacological studies have been made on CD34^+ ^stem and progenitor cells.

### Expression of P2Y receptors in lymphocytes

**Figure 1:**
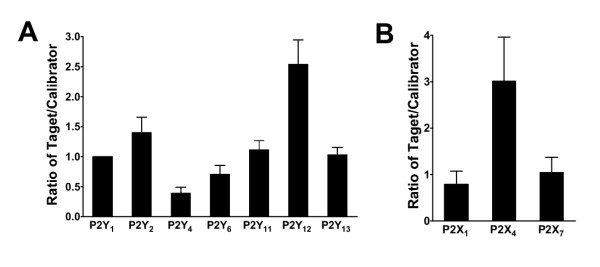
**Relative P2 gene expression in lymphocytes. **A, Bar graph shows relative P2Y_1_, P2Y_2_, P2Y_4_, P2Y_6_, P2Y_11_, P2Y_12 _and P2Y_13 _receptor gene expression normalized to GAPDH. B, Bar graph shows relative P2X_1_, P2X_4 _and P2X_7 _receptor gene expression normalized to GAPDH. P2Y_1 _was chosen to be calibrator.

In lymphocytes, all the target genes P2Y_1_, P2Y_2_, P2Y_4_, P2Y_6_, P2Y_11_, P2Y_12_, and P2Y_13 _could be detected (n = 6). To illustrate expression of the P2 receptors relative to each other the P2Y_1 _receptor was used as calibrator for the others, i. e. the other receptors were expressed as a ratio of the P2Y_1_. Among the P2Y receptor subtypes the P2Y_12 _and P2Y_2 _had highest expression (Figure 1A). The lowest expressed P2Y receptor was P2Y_4_.

Extracellular nucleotides and their P2 receptors are involved in the regulation, proliferation but also apoptosis and cell death in lymphocytes and monocytes [3,16]. Previous studies have shown that ATP, ADP, UTP and UDP stimulate phospholipase C and Ca^2+ ^release from intracellular stores, that fits well with the highly expressed P2Y_2 _receptor, together with the lesser expressed P2Y_1 _and P2Y_6 _receptors. ATP and ADP, but not UTP, can also increase cAMP [17]. This is in agreement with the P2Y_11 _receptor that had the third highest mRNA expression.

The most interesting finding was that P2Y_12 _had the highest expression among the P2Y receptors in lymphocytes. It is not likely that this is the result of platelet contamination, because platelets contain very low amounts of RNA. To the best of our knowledge, there are no studies that have examined the effects of P2Y_12 _on lymphocytes, even though selective antagonists exist. It is expected to inhibit cAMP generation and may activate lymphocytes. This could explain the antiinflammatory effect of clopidogrel. Clopidogrel is a P2Y_12 _antagonist used in the clinic as a platelet aggregation inhibitor that reduces thrombotic cardiovascular events such as myocardial infarctions. However, it has also been shown to reduce CRP, even though aspirin in antiplatelet doses lacks this effect [18]. This effect may be mediated via P2Y_12 _receptors in lymphocytes.

### Expression of P2X receptors in lymphocytes

The most abundant P2X receptor in lymphocytes was the P2X_4 _receptor. As showed in Figure 1B, the expression of P2X_4 _was 3.2 times higher than P2Y_1_. The expression of P2X_4 _was significantly higher than the expression of the other P2X receptors; P2X_1 _(P < 0.001) and P2X_7_(P < 0.01).

Selective pharmacological tools to discriminate between P2X receptors are scarce. Nevertheless, several studies have suggested the importance of P2X_7 _in lymphocyte regulation. However, B lymphocytes stimulated with ATP do not undergo the typical increase in permeability up to 900 Da that is typical for the P2X_7 _receptor. On the other hand, P2X_7 _mediated effects on Ba^2+ ^and ethidium influx, phospholipase D activity and shedding of L-selectin have been blocked by the P2X_7 _selective antagonist KN-62 in human lymphocytes [19]. Thus it is a surprising finding that the P2X_4 _receptor was the highest expressed subtype in lymphocytes at the mRNA level. Even though we have demonstrated that more than 90% of the preparation consists of lymphocytes (see methods), it is possible that a small contamination of monocytes may have influenced the results, at least regarding P2X_4 _receptor mRNA expression, because of its high expression levels in monocytes. P2X_4 _receptors have indeed been demonstrated at the protein level in human B lymphocytes by confocal immunohistochemistry, in which P2X_1_, P2X_4 _and P2X_7 _were detected at the protein level [20]. However, the P2X_4 _receptor staining was the most variable of the P2X receptors with weak to moderate levels of staining in a large proportion of cells in three patients and weak levels in only a minority of the cells from the other three patients examined [20].

### Expression of P2Y receptors in monocytes

**Figure 2:**
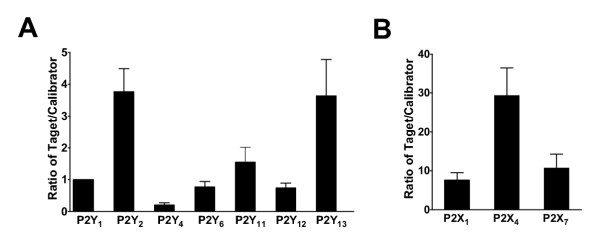
**Relative P2 gene expression in monocytes. **A, Bar graph shows relative P2Y_1_, P2Y_2_, P2Y_4_, P2Y_6_, P2Y_11_, P2Y_12 _and P2Y_13 _receptor gene expression normalized to GAPDH. B, Bar graph shows relative P2X_1_, P2X_4 _and P2X_7 _receptor gene expression normalized to GAPDH. P2Y_1 _was chosen to be calibrator.

Again, the P2Y_1 _expression was used as calibrator for the others, i. e. the other receptors were expressed as a ratio of the P2Y_1_. Among the P2Y receptors, the P2Y_2_, P2Y_13 _and P2Y_11 _had highest expression (Figure 2A, n = 6). The presence of P2Y receptor mRNA in monocytes and lymphocytes is in agreement with previous studies using regular RT-PCR [21].

Extracellular nucleotides stimulate interleukin secretion, iNOS-generation in monocytes, are involved in differentiation, cytotoxicity and killing of pathogens. All monocyte/macrophage cell lines express P2Y receptors coupled to IP_3 _generation and intracellular Ca^2+ ^release, but the individual subtypes have not been investigated in detail in monocytes [2,3]. However, both ATP and UTP are active agonists, which is in agreement with the highest mRNA expression of the ATP/UTP receptor P2Y_2 _(Fig 2). ATP mediated increase in cAMP has suggested the presence of P2Y_11_, with a suggested role in maturation of human monocyte-dendritic cells [22]. A relatively high expression of P2Y_11 _was confirmed in our experiments. Interestingly, the P2Y_13 _had even higher mRNA levels. To our knowledge, no experiments have addressed the presence of this cAMP inhibitory ADP receptor in monocytes. In fact, it has been an unresolved issue in what tissue this receptor is expressed. High levels in the spleen could be in agreement with monocyte expression [23]. Thus, the presence of P2Y_2 _and P2Y_11 _receptors are confirmed as expected, with the interesting addition of P2Y_13 _receptors. Future experiments addressing the physiological role of P2Y_13 _receptors in monocytes are needed.

### Expression of P2X receptors in monocytes

Early studies demonstrated that ATP activates a receptor on macrophages that increase cell permeability eventually leading to cell death [2,3]. P2X_7 _receptor transfection confers susceptibility to ATP-dependent permeabilization and ATP-resistant clones lack the P2X_7 _receptor, demonstrating that it is present on macrophages and necessary for permabilization. However, it is not known whether P2X_7 _is the only constitutive subunit or if it assembles with other subunits.

As showed in Figure 2B, P2X_4 _was by far the highest expressed P2 receptor in monocytes and the P2X_1 _(P < 0.01) and P2X_7 _(P < 0.01) had lower levels. Thus, unexpectedly the P2X_7 _receptor was not the highest expressed P2X receptor in monocytes. This is in agreement with patch-clamp experiments suggesting that other P2X receptors are involved [24]. Interrelation of these experiments has suggested the contribution of P2X_4 _receptors, which is supported by our findings [25]. It should be noted that all the three P2X receptors were expressed at very high levels compared to other cell types (30-fold more than the calibrator gene for P2X_4 _and 6–7-fold more for P2X_1 _and P2X_7_). A physiological role for all three subtypes can therefore be expected.

### Expression of P2 receptors in CD34^+ ^stem and progenitor cells

**Figure 3:**
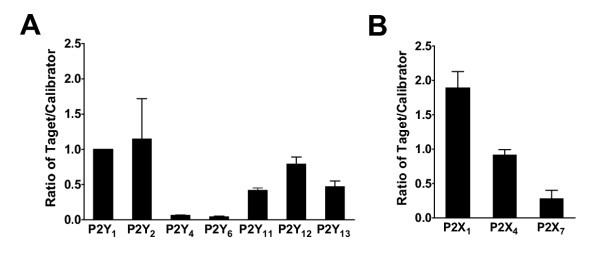
**Relative P2 gene expression in CD34^+ ^stem and progenitor cells. **A, Bar graph shows relative P2Y_1_, P2Y_2_, P2Y_4_, P2Y_6_, P2Y_11_, P2Y_12 _and P2Y_13 _receptor gene expression normalized to GAPDH. B, Bar graph shows relative P2X_1_, P2X_4 _and P2X_7 _receptor gene expression normalized to GAPDH. P2Y_1 _was chosen to be calibrator.

CD34^+ ^stem and progenitor cells are receiving an increasing attention because of their extensive self-renewal and multilineage differentiation ability making them attractive for cellular therapy [26]. Knowledge of their P2 receptor expression could be used for directing differentiation or for further subtype selection of early progenitors types. There are no previous pharmacological or expression studies of P2 receptors on human CD34^+ ^stem and progenitor cells. We found expression of several P2Y receptors, especially P2Y_1 _and P2Y_2 _(Figure 3A, n = 3). This indicates that both ATP and UTP are agonists for CD34^+ ^stem and progenitor cells and may stimulate IP_3 _and intracellular Ca^2+ ^release.

Among the P2X receptors the P2X_1 _receptor had the highest expression followed by P2X_4 _(P2X_1 _had significantly higher expression than P2X_7_, P < 0.05) (Figure 3B, n = 3), suggesting a potential role of these receptors in regulation of stem and progenitor cells. P2Y_1_, P2Y_2 _and P2X_1 _receptors have all been shown to stimulate proliferation, but also to be able to mediate apoptosis [26]. Such roles could be of major importance in the highly proliferative CD34^+ ^stem and progenitor cells. Antagonists or agonists of these receptors could be used to control their differentiation or proliferation.

## Conclusions

The P2X_4 _receptor had the highest mRNA expression in lymphocytes and monocytes. Among the P2Y receptors, P2Y_12 _and P2Y_2 _had highest expression in lymphocytes, while the P2Y_2 _and P2Y_13 _had highest expression in monocytes. The most interesting findings were the high mRNA expression of P2Y_12 _receptors in lymphocytes potentially explaining the anti-inflammatory effects of clopidogrel, P2Y_13 _receptors in monocytes and a previously unrecognised expression of P2X_4 _in lymphocytes and monocytes. In addition, for the first time P2 receptor mRNA expression patterns have been studied in CD34^+ ^stem and progenitor cells. Several P2 receptors were expressed (P2Y_2_, P2Y_1_, P2Y_12_, P2Y_13_, P2Y_11_, P2X_1_, P2X_4_), indicating a role in differentiation and proliferation. Thus, it is possible that specific antibodies to P2 receptors could be used to identify progenitors for monocytes, lymphocytes and megakaryocytes.

## Methods

The studies were approved by the local Ethics Committee of the Lund University and were conducted according to the principles of the Declaration of Helsinki.

### Preparation of monocytes and lymphocytes

Peripheral blood was drawn from each of 6 healthy volunteers (after informed consent) into heparin vials. The mononuclear cells were isolated by density gradient centrifugation on Lymphoprep™ (Axis Shield Poc AS, Oslo, Norway) at 605 g for 30 minutes. The lymphocytes and monocytes thus obtained were washed three times in RPMI 1640 medium with L-glutamine (Gibco/BRL, Life Technologies Ltd, Paisleys, Scotland) and 0.1% human serum albumin (Sigma, St Louise, MO, USA), (medium), and centrifuged each time at 605 g for 5 minutes.

The fraction of lymphocytes and monocytes obtained according to this procedure was resuspended in medium with 15% normal human serum (NHS) added to a concentration of 4 × 10^6 ^cells/ml. Flow cytometry (Epics XL-MCL Beckman-Coulter, Florida, USA) analysis on these cells by detection of cell surface CD14 and CD45 showed that approximately 10% of the cells were monocytes. 800 μl of this cell-suspension was plated on a chamber slide 4 well glass slide (Nalge Nunc International, IL, USA) at 37°C in an atmosphere containing 5% CO_2 _and 96% humidity for 1 h in order for the monocytes to adhere. Nonadherent cells were removed by washing three times with medium. Flow cytometry analysis of these nonadherent cells showed that at least 90% were lymphocytes, and were therefore used as source of lymphocytes. The cells attached to the glass slides (<90% monocytes as assessed by flow cytometry) were detached by adding first PBS and then 0.5 mM EDTA-PBS for 3 min in room temperature.

### Preparation of CD34^+ ^stem and progenitor cells

Bone marrow samples were obtained from healthy volunteers (n = 3), after informed consent, using guidelines approved by the Ethical Committee, Lund University. Mononuclear cells were isolated by density gradient centrifugation (Ficoll-Paque; Pharmacia, Uppsala, Sweden). CD34^+ ^cells were isolated by 2passages through magnetic columns (MidiMacs;Miltenyi Biotec, Bergish Gladbach, Germany) by using a hapten-conjugated CD34 antibody (Qbend/10) and an antihapten antibody conjugated to magnetic beads (CD34^+ ^isolation kit; Miltenyi Biotec). CD34 expression was analyzed by immunostaining with a FACSCalibur flow cytometer (Becton Dickinson) by using the CellQuest program (Becton Dickinson) and the purity of isolated populations was reproducibly > 95% [27].

### RNA extraction

Total cellular RNAs were extracted using TRIzol reagent (Gibco BRL, Life Technology) according to the supplier's instructions, dissolved in diethyl-pyrocarbonate (DEPC) treated water and stored at -70°C until used.

### Quantitative analysis of P2 receptors by real-time reverse transcription polymerase chain reaction

TaqMan Reverse Transcription Reagents Kit was used to transcribe mRNA into cDNA. Real-time PCR were performed by means of a PRISM 7700Sequence Detector as described previously [8,15,28,29]. Oligonucleotide primers and TaqMan probes were designed using the Primer Express software, based on sequences from the GenBank database [8,15]. Constitutively expressed GAPDH were selected as endogenous control to correct for potential variation in RNA loading or efficiency of the amplification reaction.

Previous analysis showed that amplification efficiencies were almost identical for GADPH and the following receptor mRNAs: P2Y_1_, P2Y_2_, P2Y_4_, P2Y_6_, P2Y_11_, P2Y_12_, P2Y_13_, P2X_1_, P2X_4_, and P2X_7 _normalized to GAPDH [8,15]. To confirm equal amplification efficiencies, we used the criterion of a regression slope of less than 0.1 for each gene normalized to GAPDH. This confirms that we could use the comparative C_T _method for the relative quantification of target without running standard curves on the same plate (Perkin-Elmer Applied Biosystems Inc; User Bulletin No. 2, December 1997). The amount of target and endogenous reference was determined from the comparative C_T _method. The target gene normalized to GAPDH was expressed as ΔC_T _(C_T _of target gene minus C_T _of GAPDH). P2Y_1 _was arbitrarily chosen to be the calibrator in the comparative analysis and is expressed as ΔC_TP2Y1 _(C_T _of target minus C_T _of GAPDH for P2Y_1_). The normalized calibrated value is given by the equation 2^-ΔΔCt^, where ΔΔC_T _is ΔC_T _-ΔC_TP2Y1_. To further verify the specificity of PCR assays, the PCR was performed with non-reverse-transcribed total cellular RNA and samples lacking the DNA template. No significant amplifications were obtained in any of these samples (data not shown).

### Drugs

Unless otherwise stated, all reagents and drugs were purchased from Sigma Chemical Corp, St. Louis, MI, USA. PCR consumables were obtained from Perkin-Elmer Applied Biosystems Inc, Foster City, CA, USA.

### Statistical methods

Data are expressed as mean and standard error of the mean (SEM) unless otherwise stated. n indicates the number of subjects that were tested. Statistical analysis of the normalized C_T _values (ΔC_T_) was performed with a one-way ANOVA, followed by a multiple comparison post test (Tukey's test) using GraphPad InStat version 3.00 (GraphPad Software Inc., USA). Significant differences were considered at P < 0.05 (two-tailed test).

## Authors' contributions

LW designed the study, carried out the RNA isolation and real-time PCR, and wrote the manuscript.

SEWJ supervised the isolation of CD34^+ ^stem and progenitor cells, and participated in writing the manuscript.

AB supervised the isolation of monocytes and lymphocytes, and participated in writing the manuscript.

DE conceived the study, guided throughout the study, and wrote the manuscript.

All authors read and approved the final manuscript.
